# Motor and Postural Patterns Concomitant with General Movements Are Associated with Cerebral Palsy at Term and Fidgety Age in Preterm Infants

**DOI:** 10.3390/jcm8081189

**Published:** 2019-08-08

**Authors:** Fabrizio Ferrari, Carlotta Plessi, Laura Lucaccioni, Natascia Bertoncelli, Luca Bedetti, Luca Ori, Alberto Berardi, Elisa Della Casa, Lorenzo Iughetti, Roberto D’Amico

**Affiliations:** 1 Department of Medical and Surgical Sciences of Mother, Children and Adults, University Hospital of Modena, 41124 Modena, Italy; 2 Statistic Unit, Department of Medical and Surgical Sciences, University of Modena, 41124 Modena, Italy

**Keywords:** cerebral palsy, prematurity, general movements, motor repertoire, posture, developmental trajectories

## Abstract

General movements (GMs) in combination with neurological examination and magnetic resonance imaging at term age can accurately determine the risk of cerebral palsy. The present study aimed to assess whether 11 motor and postural patterns concomitant with GMs were associated with cerebral palsy. Video recordings performed after birth in 79 preterm infants were reviewed retrospectively. Thirty-seven infants developed cerebral palsy at 2 years corrected age and the remaining 42 showed typical development. GMs were assessed from preterm to fidgety age and GM trajectories were defined. The 11 motor and postural patterns were evaluated at each age and longitudinally, alone and in combination with GM trajectories. A logistic regression model was used to assess the association between GMs, concomitant motor and postural patterns, and cerebral palsy. We confirmed that high-risk GM trajectories were associated with cerebral palsy (odds ratio = 44.40, 95% confidence interval = 11.74–167.85). An association between concomitant motor and postural patterns and cerebral palsy was found for some of the patterns at term age and for all of them at fidgety age. Therefore, at term age, concomitant motor and postural patterns can support GMs for the early diagnosis of cerebral palsy.

## 1. Introduction

Preterm newborn infants are at high risk for developmental problems. Recently, several studies have indicated a significant increase of survival among very low birth weight and extremely low birth weight infants, which has been primarily related to the continuous improvement in pre, perinatal, and neonatal care [1]. In high-income countries such as Australia and Europe, an increase in survival is accompanied by a stable or lower incidence of cerebral palsy, whereas in low-middle income countries, survival is accompanied by an increase in major and minor developmental delays [2]. According to a recent systematic review [2], cerebral palsy is the most common physical disability in childhood with a prevalence of 2.1 cases per 1000 live births in high-income countries.

Early diagnosis of cerebral palsy may lead to early intervention, which is critical to the maximum brain spurt and the greatest neuroplasticity of the third trimester after conception and of the first months after term age. It is important for clinicians in charge of neurological follow-ups (i.e., neonatologists and developmental neurologists) and it is important for parents that have to face this new challenge (i.e., the development of a small, fragile, preterm infant often affected by brain lesion), since it could result in prompt and tailored interventions aiming to limit functional impairment. General movements (GMs) assessment, in combination with Hammersmith Infant Neurological Examination and magnetic resonance imaging (MRI) at term age represent the gold standard in the diagnosis of cerebral palsy in preterm infants. The previous concept of a latent period lasting 12 to 24 months with minimal or unspecific patterns of cerebral palsy is no longer acceptable [3]. It is currently understood that cerebral palsy or a high risk of cerebral palsy can be accurately predicted before 5–6 months corrected age (CA) [2].

In the present research, we did not focus on imaging or neurological examination but rather the spontaneous motor and postural patterns that can be observed from video recordings as part of the motor repertoire between 6 and 20 weeks post-term [4]. Prechtl and colleagues have elaborated and validated the GMs method based on video recordings of spontaneous motor behaviors and on the assessment of the GM quality [5,6,7,8,9]. The new method, based on a gestalt assessment of the variability, complexity, and fluency of spontaneous motor behaviors was a great step forward for the early diagnosis of cerebral palsy. The assessment of GMs is carried out from birth up to 5 months CA [10]. Several studies have confirmed the diagnostic accuracy of GMs for cerebral palsy and three recent systematic reviews demonstrated their superior accuracy compared to other neurological diagnostic tools [2,11,12]. Several studies have also examined the developmental trajectories of GMs as predictors of cerebral palsy [7,8,13,14,15,16]. In the past, other early neurological signs such as irritability, abnormal fingers posture, spontaneous Babinski, reduced popliteal angle, retraction of the shoulders, extensor hypertonus, lower limb weakness, abnormalities of the muscle tone, jitteriness, stiffness at handling, delay in postural development, delay in the gross motor functions, and abnormal postural reactions have been described in preterm infants affected by severe neurolesions who later developed cerebral palsy [3,17,18,19,20,21]. The aforementioned neurological signs are not specific to cerebral palsy, as they are found also in transient neurological problems (i.e., transient dystonia and dissociated motor development), and other neurological disturbances [22,23]. Therefore, the role of these motor and postural signs in the diagnosis of cerebral palsy remains unclear, as well as the timing for their evaluation.

The qualitative and quantitative aspects of the early motor repertoire have been recognized as predictive of minor neurological dysfunctions at school age [24,25]. The age-adequate motor repertoire, including antigravity movements, midline leg movements and manipulation, and asymmetric tonic neck (ATN) posture has been found to be associated with lower scores of intelligence, attention, and visuo-motor integration between 5 and 7 years [24]. In the present study, we explored the possibility that a set of selected patterns from the motor repertoire in association with signs pointing to loss of balance [26] could be associated with abnormal GMs trajectories and with early signs of cerebral palsy. Among the numerous patterns of the motor repertoire recognizable at 3 to 5 months CA, we chose 11 items that in our preliminary overview of the videos appeared more frequent and typical of infants that later develop CP. We defined those as “concomitant motor and postural patterns”, since they appear at the same time as the GMs. We postulated whether these concomitant motor and postural patterns could integrate the information derived by the GMs trajectories in early recognition of cerebral palsy.

Thus, the present study aimed to answer the following questions:

(a) Which type of GM trajectories were associated with cerebral palsy in preterm infants?
(b) Which concomitant motor and postural patterns, with or without GMs, were associated with cerebral palsy at three key ages (preterm, term, and fidgety age)?
(c) Which concomitant motor and postural patterns were longitudinally considered to increase the association of GMs to cerebral palsy?

## 2. Methods

### 2.1. Study Design

This was a case-control retrospective observational study that included preterm infants born at the neonatal intensive care unit (NICU) of the University-Hospital of Modena between 1990 and 2015. The inclusion criterion was birth before 37 weeks of gestational age and the availability of at least one video at each of the ages considered. Exclusion criteria were central nervous system infections, chromosomal and genetic syndromes, and cerebral malformations. We selected two groups, one including infants developing cerebral palsy at 2 years CA and the other including infants without cerebral palsy at 2 years CA. The Provincial Ethical Committee of Modena approved the present study on 24 July 2018 and all parents provided informed consent.

### 2.2. Video Recordings

In the NICU, video recordings of spontaneous motor repertoire are typically taken at regular intervals from birth to the fidgety period for infants at risk of neurological impairment. Each video recording takes approximately 3 to 5 min. During the recording, the infant is partially dressed and in a supine position. Before recording, according to the GM assessment, the behavioral state of the infant is evaluated.

In the present study, we only considered preterm infants for which video recordings at three key ages were available: preterm (<37 weeks), term (37–42 weeks), and fidgety (51–56 weeks). Three different observers, who were not aware of the presence/absence of cerebral palsy, assessed 3 min of optimal recording for each age. The mean inter-scorer agreement was 90.3%. In case of disagreement among them, the videos were re-evaluated, and consensus was reached by discussion.

### 2.3. Evaluation of Spontaneous Motor Repertoire

The observers evaluated the quality of the GMs according to Prechtl’s method along with 11 motor and postural pattern such as: signs pointing to loss of balance, tremors, movements towards midline in upper limbs, movements towards midline in lower limbs, open hand posture, finger spreading, monotonous/stereotyped limbs movements, asymmetric tonic neck posture, head posture on a side, pathological head movements, and asymmetries.

GMs are gross movements involving the whole body, which are observable from the fetal period until approximately 5 months post-term age. They change their characteristics over time, according to gestational age. GMs present themselves as movements with variable speed, force, and intensity involving the whole body with a gradual beginning and ending. From birth to 46 weeks postmenstrual age (PMA), GMs are called writhing movements. Between 46 and 50 weeks PMA writhing movements gradually are replaced by fidgety movements (FMs), which are characterized by small amplitude, moderate speed, and variable acceleration. FMs are observed when infants are awake and not crying [10].

In the present study, the quality of writhing movements was scored as normal (N), poor repertoire (PR), or cramped-synchronized (CS), whereas FMs were scored as F+ when they were continuous or intermittent or F− when they were absent or sporadic.

Regarding the 11 concomitant motor and postural patterns, we considered 10 patterns referring to the assessment of motor repertoire [4] and an additional one of “signs pointing to loss of balance”, which was observed by Ferrari et al. [26] in a study on the normal motor and postural behavior in a group of 21 low-risk infants followed from 30–33 to 46–54 weeks PMA. The detailed scoring system is shown in Table 1.

### 2.4. Statistical Methods

Statistical analyses were performed using SPSS for Windows (version 22.0). GM trajectories were defined by dividing the observational period in halves: the first part corresponding to the period from birth to term age and the second one referring to fidgety age. In the first period, the following five GM trajectories were identified:

1. Normal trajectory, when normal GMs were present at both preterm and term age (N-N);
2. Transient poor repertoire trajectory, when poor repertoire GMs were present at either preterm or term age (PR-N or N-PR);
3. Persistent poor repertoire trajectory, when poor repertoire was present at both preterm and term age (PR-PR);
4. Transient cramped-synchronized trajectory, when cramped-synchronized was present at either preterm or term age (CS-PR or PR-CS);
5. Persistent cramped-synchronized trajectory, when cramped-synchronized was present at both preterm and term age (CS-CS).

At fidgety age, three of these trajectories (2, 3, 4) were classified according to the presence or absence of fidgety movements resulting in eight final trajectories:

1. N-N, F+
2. PR-N, F+ or N-PR, F+
3. PR-N, F− or N-PR, F−
4. PR-PR, F+
5. PR-PR, F−
6. CS-PR, F+ or PR-CS, F+
7. CS-PR, F− or PR-CS, F−
8. CS-CS, F−

A univariate logistic regression analysis was then performed to evaluate the association between each of the eight GM trajectories and the motor outcome at 2 years CA. Based on the results of univariate analysis, we identified high and low risk GM trajectories in preterm infants.

A univariate logistic regression analysis was performed to evaluate the association between concomitant motor and postural patterns and the motor outcome at 2 years CA, regardless of GMs. Odds ratio (OR) was used as a measure of association and was reported along with its 95% confidence intervals (95% CI). In case of sparse data, ORs were estimated using exact logistic regression. The identification of a set of concomitant motor and postural patterns associated with cerebral palsy was executed using a forward stepwise regression. Trajectories of concomitant motor and postural patterns associated with cerebral palsy in preterm infants were identified to describe how the change of these items over time affects the outcome. Each trajectory was considered at high or low risk. This classification was initially based on clinical assessment and then statistically evaluated using logistic regression analysis. These trajectories are reported in Table 2.

The extent to which any trajectory increased the diagnostic accuracy of GM trajectories in predicting cerebral palsy was estimated by sensitivity and specificity. These measures were reported along with their 95% CIs, calculated by using Clopper-Pearson method.

## 3. Results

Thirty-seven preterm infants with cerebral palsy and 42 without cerebral palsy were enrolled. Clinical characteristics are shown in Table 3.

Clinical characteristics of the two groups were compared using the Mann–Whitney U test for continuous variables and Fisher’s exact test for categorical ones. The two groups differed with respect to 1 min Apgar score and ultrasonographic cerebral lesions.

### 3.1. GM Trajectories Associated with Cerebral Palsy in Preterm Infants

The association between GM trajectories and cerebral palsy is reported in Table 4. Two infants were not included in the analysis, as videos were not available.

Trajectories differed between the two groups, as seen in Table 4. Among the eight possible GM trajectories, three were observed only in the control group (normal GMs and F+, transient poor repertoire and F+, transient cramped-synchronized and F+) and two (transient cramped-synchronized and F−, persistent cramped-synchronized and F−) only in the case group. Two trajectories (persistent poor repertoire and F+, persistent poor repertoire and F−) were seen in both groups and one was never observed (transient poor repertoire and F−).

**Figure 1:**
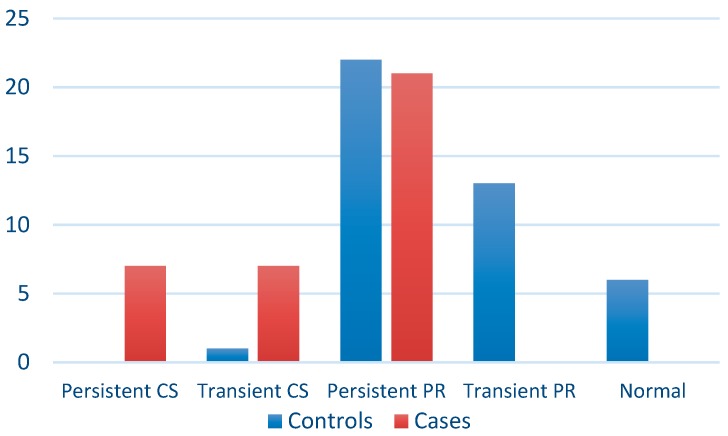
GMs trajectories. N = normal; CS = cramped-synchronized; PR = poor repertoire.

Trajectories with cramped-synchronized followed by F− were significantly associated with cerebral palsy, whereas persistent poor repertoire trajectories, which were the most frequent in both groups (Figure 1), were not.

The GMs trajectories were classified as high and low risk. The high risk ones included persistent poor repertoire GMs and transient and persistent cramped-synchronized GMs trajectories not followed by FMs, whereas the low risk ones included the remaining trajectories. The association between high and low risk GM trajectories and cerebral palsy was found to be statistically significant (OR = 44.40, *p* < 0.001; Table 5).

### 3.2. Concomitant Motor and Postural Patterns Associated with Cerebral Palsy Regardless of GMs in Preterm Infants

The association between concomitant motor and postural patterns and cerebral palsy at the three key ages is shown in Table 6.

The analysis at the three key ages showed:

(a) At preterm age, only two patterns were associated with cerebral palsy: open hand posture and absence of signs pointing to loss of balance.
(b) At term age, the following seven patterns were associated with cerebral palsy: tremors, movements towards midline in upper limbs (few or absent), movements towards midline in lower limbs (absent), open hand posture, monotonous/stereotyped limbs movements, head posture on a side, and pathological head movements.
(c) At fidgety age, all concomitant motor and postural patterns were associated with cerebral palsy.

### 3.3. Concomitant Motor and Postural Patterns That, along with GMs, were Associated with Cerebral Palsy at the Three Key Ages

The association between GMs along with each concomitant motor and postural pattern and cerebral palsy was assessed. The following concomitant motor and postural patterns in combination with GMs were found associated with cerebral palsy: at preterm age, signs pointing to loss of balance and open hand posture; at term age, tremors, open hand posture, and monotonous/stereotyped limbs movements; and at fidgety age, signs pointing to loss of balance, tremors, movements towards midline in lower limbs, finger spreading, monotonous/stereotyped limbs movements, asymmetric tonic neck posture, and asymmetries.

Table 7 demonstrates reported sets of concomitant motor and postural patterns that, in combination with GMs, are mostly associated with cerebral palsy. At preterm age, absence of signs pointing to loss of balance and open hand posture with abnormal GMs were the patterns that were most often associated with cerebral palsy. At term age, predominantly open hand posture and monotonous stereotyped limbs movements along with abnormal GMs were the patterns that were most often associated with cerebral palsy. Finally, at fidgety age, monotonous/stereotyped limbs movements along with abnormal GMs were the patterns that were primarily associated with cerebral palsy.

Complete results can be found in Appendix A (Table A1 and Table A2).

### 3.4. Concomitant Motor and Postural Pattern Trajectories Associated with Cerebral Palsy

**Figure 2:**
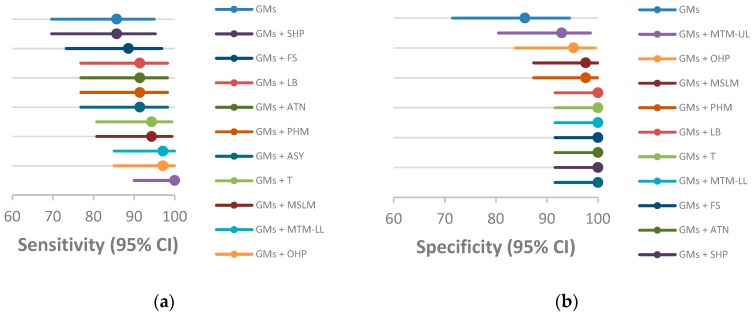
Sensitivity (**a**) and specificity (**b**) of GMs trajectories alone and in combination with concomitant motor and postural patterns trajectories. LB = signs pointing to loss of balance, T = tremors, MTM-UL = movements towards midline in upper limbs, MTM-LL = movements towards midline in lower limbs, OHP = open hand posture, FS = finger spreading, MSML = monotonous/stereotyped limbs movements, ATN = asymmetric tonic neck posture, HPS = head posture on a side, PHM = pathological head movements, ASY = asymmetries.

The diagnostic accuracy of each trajectory is reported in Table 8. All showed a high level of sensitivity and specificity. Sensitivities and specificities of all trajectories, including any of concomitant motor and postural patterns were higher than those observed in the trajectory based only on GMs, although their CIs overlap (Figure 2).

## 4. Discussion

The American Academy for Cerebral Palsy and Developmental Medicine [27] recommends developing all possible strategies to reach an early identification of cerebral palsy to commence targeted goal-directed appropriate interventions as early as possible. Along with this recommendation, we considered GMs and some motor and postural patterns from the motor repertoire, defined as “concomitants”, in two groups of preterm infants, one developing cerebral palsy and one without. Both groups were followed with video recordings at preterm, term, and fidgety age. The indications that emerged from the preterm period were few: predominant open hand posture and a delay in signs pointing to loss of balance were the only two patterns that differed when comparing the two groups of preterm newborn infants. Contrarily, findings from term and fidgety age were numerous and supportive of the possible diagnosis of cerebral palsy and the need of early intervention. Term and fidgety age are two critical periods for later development: a large portion of preterm infants with poor repertoire GMs at preterm age tend to normalize at term or fidgety age. The earlier the normalization, the better the outcome [8].

Contemporaneous to the period of GM window is the development of the motor repertoire [10] that consists of movements and postural patterns other than GMs that point to the emergence of new goal-directed antigravity movements. The coexistence at term age of one or more concomitants with abnormal GMs indicates a neurological dysfunction that might evolve to cerebral palsy. Our present study demonstrated that most of the concomitants appear at term age; the more numerous they are at term and the more they persist at fidgety age, the more likely the evolution to cerebral palsy.

It is known that serial GM assessments in preterm infants provide a more accurate prognosis than a single assessment that can be influenced by transient clinical conditions [28,29] or by drugs primarily used to control clinical respiratory problems (e.g., post-birth corticosteroids, caffeine, analgesics, etc.). Nevertheless, there is a paucity of studies on the evolution of GMs trajectories in preterm infants and even fewer are the studies with serial GM assessments starting early after birth and extending up to the fidgety age [16,30,31,32,33,34,35,36,37,38]. Our present study suggests that among the eight different possible types of trajectories, only two were associated with cerebral palsy and included in both instances was cramped-synchronized, either transient or persistent, followed by the absence of fidgety movements. The predictive power of cramped-synchronized GMs was already determined by previous studies. Ferrari et al. [7] demonstrated that in preterm infants the cramped-synchronized trajectory of GMs, if persistent and/or predominant during the GMs window, was predictive of cerebral palsy. The same authors [39] demonstrated that the earlier the cramped-synchronized GM appearance, the worse the functional impairment at two years CA. In the present study, only cramped-synchronized GMs trajectories were associated with cerebral palsy at 2 years CA. Poor repertoire GMs were not significantly associated with CP; however, it is wise to consider poor repertoire GMs with caution. Poor repertoire GMs are the most common motor abnormality and when it is persistent, may preface a normal outcome, minor developmental problems, or cerebral palsy. Moreover, at term age, poor repertoire GMs may be difficult to distinguish from normal GMs because the writhing and repetitive sequencing of limb movements present in normal GMs may mimic poor repertoire GMs.

This is why it is imperative, especially at term age, to search for other motor and postural patterns that can support and refine the prevision of cerebral palsy. In the present study, concomitant motor and postural patterns were assessed alone or in combination with GMs at three key ages. At preterm age, two concomitant motor and postural patterns were associated with cerebral palsy, namely open hand posture and absence of signs pointing to loss of balance. At term age, there were seven concomitant motor and postural patterns associated with cerebral palsy: tremors, few or absent movements towards the midline in upper limbs, absent movements towards the midline in lower limbs, open hand posture, monotonous/stereotyped limbs movements, head on a side, and pathological head movements. At fidgety age, all concomitant motor and postural patterns were associated with cerebral palsy and the extent of this association increased compared with term age. In addition to the seven concomitant motor and postural patterns seen at term age, three new patterns were present: finger spreading, frequent/dominant asymmetric tonic neck posture, and asymmetry between postures and movements of the two body sides.

We also investigated which individual concomitant motor and postural patterns, among those previously mentioned, when combined with GMs were associated with cerebral palsy. The concomitant motor and postural patterns that allow additional information to GMs were, at preterm age, signs pointing to loss of balance and open hand posture; at term age, tremors, open hand posture, and monotonous/stereotyped limbs movements; and at fidgety age signs pointing to loss of balance, tremors, movements towards the midline in lower limbs, finger spreading, monotonous/stereotyped limbs movements, asymmetric tonic neck posture, and asymmetries.

We also identified sets of concomitant motor and postural patterns that, in combination with GMs, were associated with cerebral palsy. These sets included: at preterm age, abnormal GMs, open hand posture and absence of signs pointing to loss of balance; at term age, abnormal GMs, open hand posture and monotonous/stereotyped limbs movements; at fidgety age, abnormal GMs and monotonous/stereotyped limbs movements.

Finally, concomitant motor and postural patterns were longitudinally evaluated, and developmental trajectories were also defined for GMs. All the trajectories in association with GM trajectories demonstrated increased sensitivity and/or specificity in the diagnosis of cerebral palsy.

### Limitations

This is a retrospective study, with infants observed and video recorded starting from the early nineties and the first two decades of the 2000s. During the last two to three decades, the frequency of the most severe hemorrhagic and ischemic lesions have diminished [40,41]. During the same period, there have been countless changes in the NICU and neonatology departments for developmental care and timing of rehabilitative interventions. In particular, motor and postural support and family involvement in multisensory stimulation of preterm infants starting in the early weeks of life have become increasingly common. The reduction of pain and stress have become a priority in the developmental care policy in the NICU and the neonatology departments. These changes have taken place progressively; therefore, we must consider that patients born in three different decades have received different care. Another limitation is related to the fact that the list of 11 motor and postural patterns is new and needs to be validated. Future prospective multicenter studies should address these limitations and possibly consider groups of preterm infants exposed to similar care strategies.

## 5. Conclusions

Concomitant motor and postural patterns evaluated both at key ages (preterm, term, fidgety) and longitudinally as trajectories are an important integration of GMs for the early diagnosis of cerebral palsy. It is important to observe the quality of GMs early, possibly at preterm age. At term age, the majority of the concomitants are present and can indicate a trend towards cerebral palsy. Therefore, if the GM trajectory is poor repertoire since preterm and term age, it is imperative to search for the concomitant motor and postural patterns, either alone or associated in clusters. In this second instance, they will define a syndromic picture that is likely to lead to cerebral palsy.

## Figures and Tables

**Table 1:** Concomitant motor and postural patterns.

Patterns	Definition	Score
Signs pointing to loss of balance	Presence of at least one minor and one major sign. Major signs are sudden abduction-extension of the four limbs, burst of tremors, abrupt rolling to the side, abrupt limb movements and/or head rotation at lifting of hips, burst of fast movements at three or four limbs lifting, side-to-side oscillation of lower limbs. Minor signs are finger spreading, abrupt abduction-extension of arms.	0. Absent 1. Present
Tremors	Involuntary, rhythmical oscillatory movement of equal amplitude around a fixed axis.	0. Absent 1. Some 2. Many
Movements towards midline in upper limbs	Antigravity movements in which a part of upper limbs moves towards or exceeds the midline (ideally considered as a line from the vertex to the lower extremities in supine position) resulting in contacts between two body parts. We distinguished simple contacts (many or some) from manipulation.	0. Manipulation 1. Many contacts 2. Some contacts 3. Absent
Movements towards midline in lower limbs	Antigravity movements in which a part of lower limbs moves towards or exceeds the midline (ideally considered as a line from the vertex to the lower extremities in supine position) resulting in contacts between two body parts. We distinguished simple contacts (many or some) from manipulation.	0. Manipulation 1. Many contacts 2. Some contacts 3. Absent
Open hand posture	Open hand posture mainly maintained throughout the observation.	0. Absent 1. Present
Finger spreading	Abrupt unilateral or bilateral full or partial extension of fingers.	0. Absent 1. Present
Monotonous/stereotyped limbs movements	Repetition of the same limbs’ movements over time. Examples are: - Stereotyped/monotonous kicking; - Rowing movements of upper limbs; - Pedaling movements of lower limbs.	0. Absent 1. Present
Asymmetric tonic neck posture	Face turned to one side with extended arm and leg on the same side and flexed arm and leg on the opposite side.	0. Absent 1. Occasional 2. Frequent/dominant
Head posture on a side	Head posture mainly maintained throughout most of the observation.	0. Absent 1. Present
Pathological head movements	Repetitive side-to-side head movements, and/or neck hyperextension.	0. Absent 1. Present
Asymmetries	Lack of equality in posture or movements between the two body sides.	0. Absent 1. Present

**Table 2:** Concomitant motor and postural patterns’ trajectories.

Patterns	Trajectory	Description
Signs pointing to loss of balance	*Low risk*	Signs pointing to loss of balance disappearing before fidgety age
	*High risk*	Patterns pointing to loss of balance still present at fidgety age
Tremors	*Low risk*	Tremors disappearing before fidgety age
	*High risk*	Tremors still present at fidgety age
Movements towards midline in upper limbs	*Low risk*	Manipulation appears at fidgety age. When there is no manipulation at fidgety age, many contacts on the midline must be present starting from term age
	*High risk*	Occasional movements towards midline at term age and no manipulation at fidgety age
Movements towards midline in lower limbs	*Low risk*	Manipulation appearing at fidgety age. When there is no manipulation at fidgety age, many contacts on the midline must be present starting from term age
	*High risk*	Occasional movement towards midline at term age and no manipulation at fidgety age
Open hand posture	*Low risk*	Open hand posture disappearing before term age
	*High risk*	Open hand posture still present at term and/or fidgety age
Finger spreading	*Low risk*	Finger spreading disappearing before fidgety age
	*High risk*	Finger spreading still present at fidgety age
Monotonous/stereotyped limbs movements	*Low risk*	Monotonous or stereotyped limbs movements disappearing before term age
	*High risk*	Monotonous or stereotyped limbs movements still present at term and/or fidgety age
Asymmetric tonic neck (ATN) posture	*Low risk*	ATN posture disappearing or becoming occasional at fidgety age
	*High risk*	ATN posture frequent or dominant at fidgety age
Head posture on a side	*Low risk*	Head-on-midline posture predominant at fidgety age
	*High risk*	Head posture on a side predominant at fidgety age
Pathological head movements	*Low risk*	Normal head movements present from term age
	*High risk*	Side-to-side head movements or neck hyperextension present at term and/or fidgety age
Asymmetries	*Low risk*	Asymmetric postures and movements disappearing before fidgety age
	*High risk*	Asymmetric postures and movements present at fidgety age

**Table 3:** Clinical characteristics.

Clinical Characteristics		Cases *N* = 37	Controls *N* = 42	*p*
**Infant Characteristics at Birth**				
Male/female ratio		16:21	14:28	0.368
Birth gestational age in weeks (mean ± SD ^1^)		28.4 ± 3	28.7 ± 2	0.395
Birth weight (mean ± SD)		1148 ± 499	1065 ± 240	0.694
Birth head circumference (mean ± SD)		26 ± 3	26 ± 2	0.579
Birth weight for gestational age	*Number of SGA* ^2^ *infants (%)*	2 (5.4%)	10 (23.8%)	0.080
	*Number of AGA* ^3^ *infants (%)*	29 (78.4%)	26 (61.9%)	
	*Number of LGA* ^4^ *infants (%)*	6 (16.2%)	6 (14.3%)	
Apgar score	*Number of infants with 1*-*min Apgar score < 7 (%)*	27 (73.0%)	19 (45.2%)	0.005
	*Number of infants with 5*-*min Apgar score < 7 (%)*	12 (32.4%)	8 (19.0%)	0.122
US cerebral lesions				
Number of IVH ^5^ 1 (%)		2 (5.4%)	1 (2.4%)	<0.001
Number of IVH 2 (%)		10 (27.0%)	1 (2.4%)	
Number of IVH 3 (%)		8 (21.6%)	0 (0.0%)	
Number of IVH 4 (%)		4 (10.8%)	0 (0.0%)	
Number of PVL ^6^ 1 (%)		8 (21.6%)	12 (28.6%)	0.001
Number of PVL 2 (%)		4 (10.8%)	5 (11.9%)	
Number of PVL 3 (%)		8 (21.6%)	0 (0.0%)	
Number of PVL 4 (%)		4 (10.8%)	0 (0.0%)	

^1^ SD = standard deviation, SGA2 = small for gestational age, ^3^ AGA = adequate for gestational age, ^4^ LGA = large for gestational age, ^5^ IVH = intraventricular hemorrhage, ^6^ PVL = periventricular leukomalacia.

**Table 4:** General Movements (GMs) trajectories associated with Cerebral Palsy.

Trajectories	Group				OR	95% CI	*p*
	Control *N* = 42		Case *N* = 42				
	*n*	%	*n*	%			
Normal GMs and F+	6	14.3	0	0	Ref.	-	-
Transient PR and F+	14	33.3	0	0	2.23	0.04–125.22	0.6962
Persistent PR and F+	16	38.1	5	13.5	4.33	0.21–90.05	0.3435
Transient CS and F+	1	2.4	0	0	4.33	0.03–560.31	0.5545
Persistent PR and F−	5	11.9	16	43.2	39.00	0.67–2257.11	0.0768
Transient CS and F−	0	0	7	18.9	195.00	3.37–11,285.55	0.0109
Persistent CS and F−	0	0	7	18.9	195.00	10.65–3569.68	0.0004

*N* = number of infants, CS = cramped-synchronized, PR = poor repertoire, F+ = continual or intermittent fidgety, F− = absent and sporadic fidgety.

**Table 5:** Low/high risk GMs trajectories and neurological outcome.

Trajectories	Group				OR	95% CI	*p*
	Control		Case				
	*n*/*N*	%	*n*/*N*	%			
Low risk	37	88.1	5	13.5	-	-	-
High risk	5	11.9	30	81.1	44.40	11.74–167.85	<0.001

**Table 6:** Association between concomitant motor and postural patterns and cerebral palsy at the three key ages.

Patterns		Odds Ratio (95% CI)		
		Preterm Age	Term Age	Fidgety Age
**LB**		0.23 (0.09–0.62)	0.59 (0.23–1.48)	20.90 (1.15–379.98) *
T		0.80 (0.25–2.55)	5.19 (1.87–14.43)	46.84 (2.67–822.91) *
MTM-UL	*Many*	-	-	13.89 (2.90–66.62)
	*Some*	-	5.20 (1.66–16.26)	16.67 (3.84–72.33)
	*Absent*	2.56 (0.77–8.53)	12.00 (1.84–78.37)	22.22 (3.72–132.75)
MTM-LL	*Many*	-	-	14.88 (1.75–126.50)
	*Some*	-	2.22 (0.52–9.54)	27.20 (2.71–272.83)
	*Absent*	1.04 (0.42–2.55)	6.06 (1.37–26.76)	59.50 (5.95–595.04)
OHP		3.83 (1.49–9.89)	7.81 (2.51–24.30)	9.57 (1.12–81.93)
FS		0.34 (0.10–1.11)	0.49 (0.15–1.66)	17.54 (0.95–322.99)
MSLM		3.25 (0.77–13.68)	12.50 (4.14–37.74)	175.71 (20.52–1504.80)
ATN		1.85 (0.53–6.45)	2.44 (0.93–6.39)	10.85 (3.47–33.98)
HPS		1.88 (0.57–6.12)	2.67 (1.07–6.64)	6.43 (1.29–32.05)
PHM		5.29 (0.56–49.71)	15.19 (1.84–125.53)	5.50 (1.40–21.64)
ASY		2.49 (0.22–28.62)	1.14 (0.07–18.87)	7.04 (1.82–27.28)

LB = signs pointing to loss of balance, T = tremors, MTM-UL = movements towards midline in upper limbs, MTM-LL = movements towards midline in lower limbs, OHP = open hand posture, FS = finger spreading, MSML = monotonous/stereotyped limbs movements, ATN = asymmetric tonic neck posture, HPS = head posture on a side, PHM = pathological head movements, ASY = asymmetries, * = in case of sparse data, the odds ratios were estimated by using exact logistic regression. The odds ratio (OR) of PHM at preterm age is high but not significant, probably in relation to the small number of patients with this pattern in both group.

**Table 7:** Concomitant motor and postural patterns that along with GMs increase their association with cerebral palsy.

Age	Pattern		OR	95% CI
**Preterm age**	GMs	Normal	reference	reference
		PR	15.60	0.87–280.15
		CS	323.00	5.75–18131.35
	LB	Absent	reference	reference
		Present	0.23	0.09–0.62
	OHP	Absent	reference	reference
		Present	3.83	1.49–9.89
Term age	GMs	Normal	reference	reference
		PR	33.00	1.87–581.28
		CS	178.20	7.87–4037.41
	MSLM	Absent	reference	reference
		Present	12.50	4.14–37.74
	OHP	Absent	reference	reference
		Present	7.81	2.51–24.30
Fidgety age	GMs	F +	reference	reference
		F −	47.36	12.57–178.51
	MSLM	Absent	reference	reference
		Present	175.71	20.52–1504.80

PR = poor repertoire, CS = cramped-synchronized, LB = signs pointing to loss of balance, OHP = open hand posture, MSML = monotonous/stereotyped limbs movements.

**Table 8:** Sensitivity and specificity if we consider GMs trajectories alone and in association with trajectories of concomitant motor and postural patterns.

	Sensitivity % (95% CI)	Specificity % (95% CI)
GMs trajectories	85.7 (69.7–95.2)	85.7 (71.5–94.6)
GMs trajectories + LB trajectories	91.4 (76.9–98.2)	100 (91.6–100 *)
GMs trajectories + T trajectories	94.3 (80.8–99.3)	100 (91.6–100*)
GMs trajectories + MTM–UL trajectories	100 (90.0–100 *)	92.9 (80.5–98.5)
GMs trajectories + MTM–LL trajectories	97.1 (85.1–99.9)	100 (91.6–100 *)
GMs trajectories + OHP trajectories	97.1 (85.1–99.9)	95.2 (83.8–99.4)
GMs trajectories + FS trajectories	88.6 (73.3–96.8)	100 (91.6–100 *)
GMs trajectories + MSLM trajectories	94.3 (80.8–99.3)	97.6 (87.4–99.9)
GMs trajectories + ATN trajectories	91.4 (76.9–98.2)	100 (91.6–100 *)
GMs trajectories + HPS trajectories	85.7 (69.7–95.2)	100 (91.6–100 *)
GMs trajectories + PHM trajectories	91.4 (76.9–98.2)	97.6 (87.4–99.9)
GMs trajectories + ASY trajectories	91.4 (76.9–98.2)	100 (91.6–100 *)

LB = signs pointing to loss of balance, T = tremors, MTM-UL = movements towards midline in upper limbs, MTM-LL = movements towards midline in lower limbs, OHP = open hand posture, FS = finger spreading, MSML = monotonous/stereotyped limbs movements, ATN = asymmetric tonic neck posture, HPS = head posture on a side, PHM = pathological head movements, ASY = asymmetries, * = one-sided, 95.7% CI.

## Appendix A

**Table A1:** Statistical characteristics of models used to identify individual concomitant motor and postural patterns that along with GMs are mostly associated with cerebral palsy.

Age	Model	−2 Log-Likelihood	ΔLL	Degrees of Freedom	Δdf	p
**Preterm age**	GMs	82.577	-	2	-	<0.001
	GMs + LB	76.245	6.332	3	1	0.012
	GMs + T	82.102	0.474	3	1	0.491
	GMs + MTM-UL	79.827	2.749	3	1	0.097
	GMs + MTM-LL	82.216	0.361	3	1	0.548
	GMs + OHP	74.523	8.054	3	1	0.005
	GMs + FS	79.720	2.857	3	1	0.091
	GMs + MSLM	80.562	2.015	3	1	0.156
	GMs + ATN	81.437	1.140	3	1	0.286
	GMs + HPS	80.216	2.361	3	1	0.124
	GMs + PHM	82.556	0.021	3	1	0.885
	GMs + ASY	82.556	0.021	3	1	0.885
**Term age**	GMs	78.878	-	2	-	<0.001
	GMs + LB	76.318	2.005	3	1	0.157
	GMs + T	73.680	4.642	3	1	0.031
	GMs + MTM-UL	73.027	5.295	4	1	0.071
	GMs + MTM-LL	73.681	4.642	4	1	0.098
	GMs + OHP	66.528	11.794	3	1	0.001
	GMs + FS	76.732	1.590	3	1	0.207
	GMs + MSLM	63.337	14.985	3	1	<0.001
	GMs + ATN	75.938	2.384	3	1	0.123
	GMs + HPS	77.795	0.527	3	1	0.468
	GMs + PHM	76.668	1.654	3	1	0.198
	GMs + ASY	77.956	0.366	3	1	0.545
**Fidgety age**	GMs	59.968	-	1	-	<0.001
	GMs + LB	49.048	10.920	2	1	0.001
	GMs + T	55.225	4.743	2	1	0.029
	GMs + MTM-UL	52.257	7.712	4	1	0.052
	GMs + MTM-LL	45.225	14.743	4	1	0.002
	GMs + OHP	58.950	1.018	2	1	0.313
	GMs + FS	53.952	6.016	2	1	0.014
	GMs + MSLM	34.729	25.239	2	1	<0.001
	GMs + ATN	51.601	8.367	2	1	0.004
	GMs + HPS	58.945	1.024	2	1	0.312
	GMs + PHM	57.004	2.964	2	1	0.085
	GMs + ASY	53.578	6.390	2	1	0.011

LB = signs pointing to loss of balance, T = tremors, MTM-UL = movements towards midline in upper limbs, MTM-LL = movements towards midline in lower limbs, OHP = open hand posture, FS = finger spreading, MSML = monotonous/stereotyped limbs movements, ATN = asymmetric tonic neck posture, HPS = head posture on a side, PHM = pathological head movements, ASY = asymmetries.

**Table A2:** Statistical characteristics of models used to identify sets of concomitant motor and postural patterns that along with GMs are mostly associated with cerebral palsy.

Age	Model	−2 Log-Likelihood	ΔLL	Degrees of Freedom	Δdf	p
Preterm age	GMs	82.577	-	1	-	<0.001
	GMs + OHP	74.523	8.054	2	1	0.005
	GMs + LB + OHP	68.626	5.897	3	1	0.015
Term age	GMs	81.066	-	1	-	<0.001
	GMs + MSLM	66.049	15.017	2	1	<0.001
	GMs + MSLM + OHP	54.566	11.483	3	1	0.001
Fidgety age	GMs	48.715	-	1	-	<0.001
	GMs + MSLM	34.729	13.986	2	1	<0.001

LB = signs pointing to loss of balance, OHP = open hand posture, MSML = monotonous/stereotyped limbs movements.
